# Production and optimization of polyglutamic acid from *Bacillus licheniformis*: effect of low levels of gamma radiation

**DOI:** 10.1186/s13568-025-01897-3

**Published:** 2025-06-18

**Authors:** Riham M. Youssef, Reham Samir, Ola M. Gomaa, Hala N. ElHifnawi, Mohamed A. Ramadan

**Affiliations:** 1 Department of Drug Radiation Research, National Center for Radiation Research and Technology (NCRRT), Egyptian Atomic Energy Authority, Cairo, Egypt; 2 Department of Microbiology and Immunology, Faculty of Pharmacy, Cairo University, Kasr El‑Aini St., Cairo, 11562 Egypt; 3 Department of Radiation Microbiology, National Center for Radiation Research and Technology (NCRRT), Egyptian Atomic Energy Authority, Cairo, Egypt

**Keywords:** Polyglutamic acid, Characterization, Polymer, *Bacilli*, Gamma radiation

## Abstract

Polyglutamic acid (PGA), has several beneficial characteristics and advantageous uses. The microbial biopolymer is released from a variety of organisms, particularly *Bacillus* species. Soil samples from various sectors of Egypt were gathered and evaluated for the production of PGA. According to the current study results, isolate (L) was the most powerful producer and was identified by 16S rRNA as *Bacillus licheniformis*. A multi-factorial Plackett–Burman layout was used to optimize PGA bacterial synthesis. Mass spectrometry, thin-layer chromatography, Fourier transform infrared spectroscopy (FTIR), and amino acid analysis were employed to characterize the product. It was also investigated how the produced polymer was affected by low gamma irradiation doses. In comparison to *Bacillus subtilis* (ATCC6633), *Bacillus licheniformis* showed an over 33% inclination in PGA yield under optimal conditions. FTIR spectra demonstrated that PGA functional groups were successfully unchanged after exposure to low levels of gamma radiation similar to the doses given to cancer patients under radiotherapy. Thermal Gravimetric Analysis (TGA) showed 2 phase weight loss for gamma irradiated PGA, emphasizing that the polymer showed thermal stability after exposure to gamma radiation. These results were confirmed by Dynamic light scattering (DLS) and liquid chromatography mass spectrometry. Finally, the isolated *Bacillus licheniformis* strain can be considered as a prospective source of PGA for future industrial and medicinal uses. Additionally, this could be a promising result for the use of PGA as a drug carrier for cancer patients undergoing radiation therapy.

**Supplementary Information:**

The online version contains supplementary material available at 10.1186/s13568-025-01897-3.

## Introduction

In contrast to other artificial non-biodegradable polymers, microbial biopolymers are becoming more and more well-liked globally since they are non-toxic, biodegradable, and environment friendly (Kreyenschulte et al. 2014). PGA is an extracellular polymer which is edible, water dissolvable, non-immunogenic, completely biodegradable and suitable for human consumption. The glutamic acid residues that make up the special anionic homopolyamide known as PGA are connected by amide bonds between amino and carboxylic acid groups (Li et al. 2022a, b). The utilization of PGA in pharmaceutical products, medicine, cosmetics, healthcare, agriculture, water treatment, and edibles is facilitated by its distinctive properties. PGA has extensive medicinal scopes including delivery mechanisms, therapeutic and immunological impacts. It has the potential to substantially lower drug toxicity and enhance drug efficacy when used in conjunction with other medications (Da Silva et al. 2014).

*Bacillus* species are employed for the safe microbial synthesis of PGA. *Bacillus* species predominantly, *B. licheniformis* and *B. subtilis* are still the most powerful producers of PGA (Liu et al. 2010; Nair et al. 2021). Glutamic acid-dependent and glutamic acid-independent producers are two categories of polyglutamic acid producers based on using an external supply of glutamic acid. The former is a promising producer because of its comparatively high PGA output; nonetheless, the cost of production is significant. However, the latter has the benefit of low production costs, but the PGA yield is constrained (Buescher and Margaritis 2007).

Some radioactive isotopes, including Cobalt 60, release short waves of extremely powerful electromagnetic radiations called gamma radiations. Gamma irradiation is the most popular cold sterilization technique for food, food packaging, and medical items. The process is simple, inexpensive, and highly effective in terms of penetrating power. Irradiation can cause the molecular weight of polymers to rise (cross-linking) or decrease (chain scission or breakdown). These effects are determined by the polymer structure and the dose delivered. All materials must be examined for the susceptibility to changes in their properties after being exposed to gamma radiation (Hara 2023).

From this perspective, the goal of our research was to select the best PGA-producing isolates and discover the critical nutrients that promote maximum production. Several factors influence PGA characteristics and yield, including the generating microbe, fermentation medium components, pH, temperature, and incubation time (Luo et al. 2016; Zhan et al. 2018; Cai et al. 2023).These factors were considered and optimized. The biosynthesized PGA was subjected to gamma radiation. Furthermore, the influence of exposure to gamma radiation at assorted doses was evaluated on the polymer properties by applying FTIR, TGA, DLS and LC–MS. As far as the authors’ know, the influence of gamma irradiation on PGA, which is concurrently provided as a medication carrier to patients undergoing radiotherapy, has not been considered before.

## Material and methods

### Soil samples collection, isolation and screening of bacteria

In sterile plastic bags, soil samples were gathered from various Egyptian governorates. Five milliliters of sterile broth were mixed with 1 gm of each soil sample, and the mixture was incubated for 24 h at 37 °C. After incubation, 0.1 mL of the supernatant from each tube containing the soil and culturing medium suspension was spread on nutrient agar plates. The plates were incubated at 37 °C for 24 h and checked for the presence of bacteria (Xu et al. 2005). Only mucoid colonies were chosen and isolated to assess their PGA productivity (Tork et al. 2015). The pure colonies were kept in glycerol stock at − 80 °C.

### Culture conditions for selection of PGA producing *bacilli*

The medium utilized for preliminary selection of bacterial isolates for PGA production was formed of ((g/L): glucose 10, yeast extract 5, L-glutamic acid 5, potassium dihydrogen phospate 0.5, magnesium sulphate 0.1 and agar 20). The plates were incubated at 37 °C for 24 h. The highly viscous colony was selected for PGA productivity (Xu et al. 2005).

### Identification of the isolate with highest PGA productivity

16S rRNA was used to fully identify the chosen isolate (L) after it had been tentatively identified using standard microbiological techniques. Utilizing DNA extraction kits from Zymo Research (UK) and the 16S rRNA analysis technique, the isolate was molecularly identified. Two universal primers 27F5′-AGAGTTTGATCMTGGCTCAG-3′ and 1492R 5′-GGTTACCTTGTTACGACTT- 3′ were used following the manufacturer instructions. Data were submitted to Genbank database in the National Centre for Biotechnology Information (NCBI, USA) and the DNA resemblance was evaluated using the NCBI BLAST®. The phylogenetic analysis for the isolate’s 16S rRNA gene was performed by the Neighbor-joining strategy using MEGA X software to explore the genetic linkage of the selected *Bacillus* isolate in the present work to other *Bacillus* species in the Genbank database (Kumar et al. 2018). Also, the 16S rRNA sequence was submitted to the World Data Centre for Microorganisms (WDCM) Culture Collection Ain Shams University (CCASU).

### Plackett–Burman (PB) layout for identifying the key variables affecting the output of PGA

Using a PB layout, the important parameters affecting the PGA yield were screened based on single-factor experimental results (Plackett and Burman 1946). Six factors influencing PGA production were assessed, namely, pH, glutamic acid, yeast, incubation time, temperature, and inoculum size (Table 1). For every variable, high and low scales were evaluated. The Plackett–Burman layout had 12 trials of different combinations of levels of the tested variables, each run being completed in triplicate. Following the incubation time, bacterial growth was assessed using spectrophotometry at 550 nm using UV spectrophotometer (T-60 UV–Visible Spectrophotometer, PG instruments, UK) (Tork et al. 2015).

**Table 1:** Experimental factors’ levels used for PGA production by *Bacillus subtilis* (ATCC6633) and *Bacillus licheniformis*

Factors	High level	Low level
pH	7	3
Glutamic acid (g/ L)	40	2
Yeast extract (g/ L)	40	2
Incubation time (Days)	7	3
Temperature (0C)	37	25
Inoculum size (mL)	2	0.5

### Polymer production under optimized conditions

*Bacillus licheniformis* and standard *Bacillus subtilis* (ATCC6633), which was purchased from Mircen, Cairo, Egypt (Microbiological culture collection), were grown independently in the same production environment and incubated at 37 °C for 3 days. The yield of PGA was refined using the procedure described by Goto and Kunioka (1992). PGA cell free supernatant was placed in 4L of cold ethanol and stored at 4 °C for 12 h. To separate any indissoluble contaminants, the PGA was centrifuged at 10,000 rpm and 4 °C for 30 min before being solubilized in distilled water. After 12 h of dialyzing against distilled water, the PGA solution was lyophilized (Tork et al. 2015).

## Characterization of PGA polymer

### Fourier transform infrared spectroscopy (FTIR)

Functional groups related to PGA were distinguished using Attenuated total reflectance-Fourier transform infrared spectroscopy (ATR-FTIR). Scanning of powdered PGA generated from both standard *Bacillus subtilis* (ATCC6633) and *Bacillus licheniformis*, and the standard PGA purchased from Sisco Research laboratories, India was carried out in the range of 4000–400 cm^−1^ using Bruker Vertex 70 instrument at NCRRT (National Centre for Radiation Research and Technology) (Li et al. 2022c).

### Thin layer chromatography (TLC)

For further identification of the generated PGA polymer, TLC was utilized by the subsequent procedure. The lyophilized PGA products from both* Bacilli* and the standard PGA were hydrolyzed in 7.5 M HCl. The polymer was mixed with HCl and heated at 100 °C for 24 h. To remove the residual HCl, the supernatant in the tubes was vaporized, and the hydrolyzed product was solubilized in 1 mL distilled water. Five microliters of the samples were spotted on a TLC silica plate using the solvent system of butanol/ acetic acid/ water in the proportion (4:1:3 *vlvlv*). Amino acids were revealed by spraying the separated spots on the TLC plate with 0.2% ninhydrin. The separated spots of PGA samples were compared to that of the standard PGA in color, position and shape to prove the existence of glutamic acid (Thapa et al. 2021).

### Liquid chromatography electrospray ionization tandem mass spectrometry (LC–ESI–MS)

LC–ESI–MS was employed for the analysis of pure PGA at the Faculty of pharmacy, Ain Shams University. ESI–MS positive ion acquisition mode was applied on a Xevo TQD triple quadruple Mass Spectrometer (Waters Corporation, Milford, and MA01757 U.S.A). Using ACQUITY UPLC-BEH C_18_ 1.7 µm–2.1 × 50 mm column with a flux average of 0.2 mL\min. The solvent mixture was made up of water with 0.1% formic acid and acetonitrile (Kino et al. 2009).

### Amino acid analysis

A weight of 0.1 g specimen was combined with 2.5 mL H_2_O and 2.5 mL of 6 M HCl, heated at 100 °C for 24 h and percolated by a syringe filter (0.22 µm pore size). Furthermore, 1 mL of the filtrate was evaporated to dryness, reconstituted in 0.1 M HCl and eluted by HPLC. The column temperature was maintained at 40 °C (Jajic et al. 2013).

### Irradiation source

Gamma radiation was applied at the National Centre for Radiation Research and Technology (NCRRT) on the produced PGA using ^60^Co source (Gamma cell 4000-A-India) at selected gamma radiation doses: 2, 7.5 and 70 Gy (Gray) with non-irradiated control to assess the influence of exposure to gamma radiation on the polymer characteristics. A dose rate of 0.575 kGy/h was used at the time of the experiment.

### Study of the effect of low doses gamma irradiation on PGA employing FTIR, TGA, DLS and LC–MS

FTIR was applied to characterize the bands of PGA exposed to low levels of gamma radiation. Additionally, thermal gravimetric analysis of gamma-irradiated PGA was carried out using TGA-50 Schimadzu (Japan). The temperature was increased from 0 to 700 ◦C with a heating rate of 10 °C/min and with N_2_ flow rate of 30 mL/min. The particle size distribution profile for PGA obtained from *Bacillus licheniformis* was performed for the non-irradiated polymer, and for PGA exposed to gamma radiation doses of 2, 7.5 and 70 Gy. Measurements were performed for DLS using Zeta potential/particle sizer (NICOMP380 ZLS, PSS., NICOMP particle sizing systems, Santa Barbara, California, USA) at NCRRT. The applied wavelength of the incident light was 632.8 nm from red He–Ne laser diode. Measurements were performed at 23 °C. LC–MS was employed for the analysis of PGA before and after exposure to gamma radiation applying the previously mentioned conditions.

### Statistical analysis

Minitab software (Version 18) was used for statistical analysis and graphical data presentation for the multifactorial design. Significant effects on PGA production were defined as variables with confidence levels greater than 95% (*P*-value < 0.05).

## Results

### Screening bacteria for PGA production

Eighty two soil samples were gathered from 15 governorates all over Egypt. A sum of 98 isolates of bacteria was recovered from the soil specimens. Colonies with the distinctive mucoid appearance, soft consistency, creamy white color, and irregular form of *Bacillus* species were chosen. Six collected *bacilli* isolates only exhibited high PGA yield on the selection medium plates. The (L) isolate showed the highest PGA productivity when compared to other isolates.

### Identification of the selected isolates

**Fig. 1:**
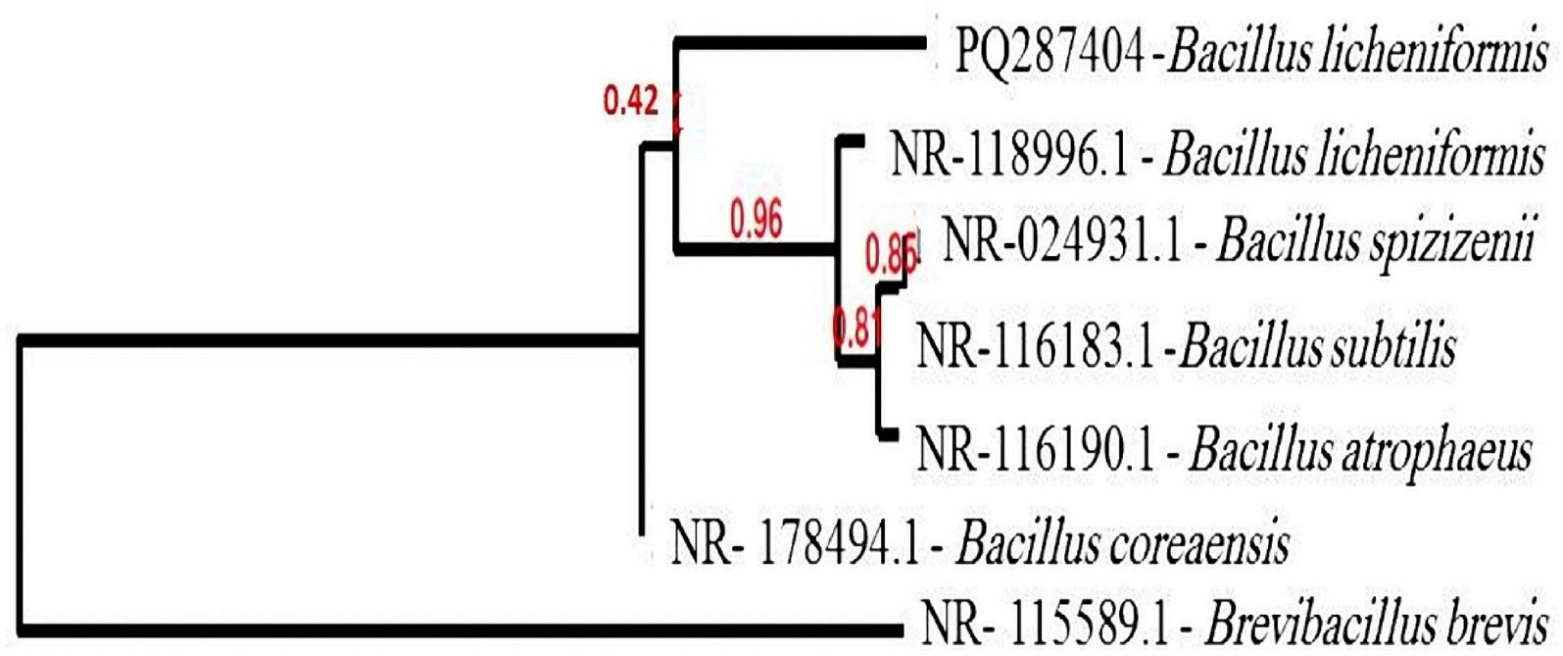
Phylogenetic tree of *Bacillus licheniformis* and related species based on 16S rRNA gene sequences. The tree was built with the MEGA X software, which employs the Neighbor-joining method

Following Gram staining, the collected isolates were categorized as either Gram positive or Gram negative bacilli. The characteristics of the (L) isolate on the culturing plate suggested that it was a member of the *Bacillus* genus. Then, it was identified on molecular basis using ribosomal nucleic acid (rRNA). Phylogenetic tree was generated using the subsequent links: www.phylogeny.fr and http://www.phylogeny.fr/simple phylogeny.cgi; as shown in Fig. 1. The isolate was submitted in Genbank as *Bacillus licheniformis* with the accession number PQ287404. Also, it was submitted to the World Data Centre for Microorganisms (WDCM) Culture Collection Ain Shams University (CCASU) (strain number, CCASU-2025-77).

### Plackett–Burman layout for variables screening and optimization

Using the Plackett–Burman (PB) experimental layout, parameters influencing the generation of PGA by *Bacillus licheniformis* and standard *Bacillus subtilis* (ATCC6633) were assessed. Minitab software (version 18) was used to perform the Plackett–Burman layout for 12 attempts with 2 concentration levels for six distinct variables. Factors combinations used in the experiments were presented in Table 2. The optical density with confidence intervals was recorded for the response of each run for both *B. licheniformis* and *B.subtilis* (ATCC 6633) as shown in Table S1 (in SI file). The maximum PGA production was obtained in trial 4 expressed in bold in Table 2 for *Bacillus subtilis* (ATCC6633) (16 g/L) and *Bacillus licheniformis* (24 g/L). The statistical examination of the variables showed that temperature, pH, glutamic acid, and incubation time had confidence level above 95% (*P* > 0.05), making them important parameters affecting the production of PGA by* Bacillus licheniformis*. Obtaining confidence levels above 95%, PGA production for the standard *Bacillus subtilis* (ATCC6633) was influenced by pH and yeast extract. At the specified culture conditions (37°C, pH = 7, inoculum size = 2 mL, incubation duration = 3 days, yeast extract 40 g/L, and glutamic acid 40 g/L), run 4 showed the highest PGA productivity.

**Table 2:** Plackett–Burman design with the observed results for the factors affecting polyglutamic acid production by both *Bacilli*

Run no	Inoculum size	Temperature	Incubation time	Yeast extract	Glutamic acid	pH	PGA *B. subtilis* (g/ L)	PGA/*B. licheniformis* (g/ L)
1	2.0	25	3	2	40	3	0.72	0.110
2	2.0	25	7	40	2	7	12.00	2.600
3	2.0	37	7	2	2	3	0.46	0.400
4	**2.0**	**37**	**3**	**40**	**40**	**7**	**16.00**	**24.000**
5	0.5	25	3	40	2	7	13.23	14.100
6	0.5	37	7	40	2	3	3.40	2.620
7	2.0	37	3	2	2	7	8.00	10.720
8	0.5	25	7	2	40	7	12.75	11.410
9	0.5	25	3	2	2	3	0.47	0.175
10	0.5	37	7	2	40	7	8.50	10.890
11	2.0	25	7	40	40	3	3.00	2.460
12	0.5	37	3	40	40	3	2.75	12.430

**Fig. 2:**
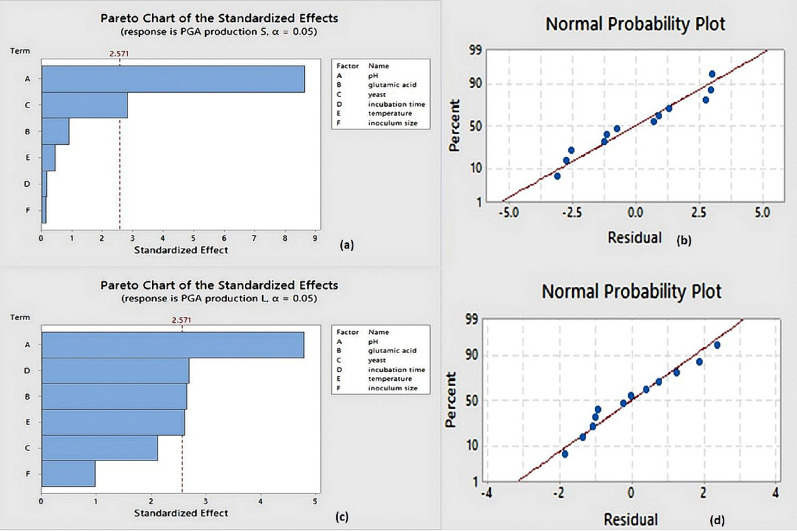
**a** Pareto chart of the variables in Plackett–Burman design of polyglutamic acid production by *Bacillus subtilis* (ATCC 6633). p values of significant factors; pH, 0.001, yeast, 0.037. **b** Normal probability plot of residuals for standard *Bacillus subtilis* (ATCC6633). **c** Pareto chart showing the variables in Plackett–Burman design for polyglutamic acid production by *Bacillus licheniformis*. Pareto charts rank the standardized effects of the examined factors. The vertical line in the chart represents a reference line; any factor that extends past this line has a significant effect at α = 0.05 (significance level). The p values of significant factors include pH (0.005), glutamic acid (0.045), incubation time (0.043), and temperature (0.048). The E-value (standardized effect of each component) was determined with Minitab software (version 18). The examined factor’s significance in influencing the response was shown by the E-value’s magnitude, and its positive or negative sign showed whether it had a positive or negative influence on the responses. **d** Normal probability plot of residuals for *Bacillus licheniformis*

The Pareto chart was recognized as a practical method of illustrating the outcomes of a Plackett–Burman layout, showing the magnitude of each variable, and assisting in the identification of the most significant variables. On a standardized Pareto chart, the longitude of each bar in this chart corresponded to the absolute value of the regression coefficient or estimated effect that goes with it. Employing Minitab software (version 18), the standardized effect of each factor (E-value) was obtained. The E-value magnitude indicated the tested factors effect in influencing the response. Pareto charts demonstrated the significance of each factor’s standardized impacts (Fig. 2a and c).

$$ {\text{R}}^{{2}} = {9}0.{83}\%;\quad {\text{adjR}}^{{2}} = {79}.{83}\%. $$

The model’s validity was examined using the determination coefficient (R^2^). R^2^ value of 0.9083 was determined for *Bacillus licheniformis*, which means that model could account for 90.83% of the response’s overall variability and just 9.17% of it was not explained. R^2^ closed to 1.0 indicates a high correlation in a regression model (Mabrouk et al. 2012). Consequently, the current R^2^-value indicated that the model is trustworthy for predicting PGA yield and showed an excellent fit between the observed and expected responses.

Additionally, the adjusted determination coefficient value (Adj R^2^ = 0.7983) validated the model’s significance. The polynomial model equation suggested to estimate the ideal levels of the variables for PGA yield after the ANOVA statistical test was done, revealed that, the first order models for PGA production were satisfactory. The equation could be written as:

$$ \begin{aligned} \begin{aligned} {\text{PGA}}\;{\text{production}}\;{\text{L}} = - 13.93 + 2.314\;{\text{pH}} + 0.1346\;{\text{glutamicacid}} \\  \quad + 0.1075{\text{yeast}} - 1.298\;{\text{incubation}}\;{\text{time}} \\  \quad + 0.420\;{\text{temperature}} - 1.26\;{\text{inoculum}}\;{\text{size}} \\ \end{aligned} \\ \end{aligned} $$

R^2^ value of 0.9437 was obtained for *Bacillus subtilis* (ATCC6633), this model was able to explain 94.37% of the response’s overall variability, leaving only 5.63% of it was unexplained. Additionally, the adjusted determination coefficient value (Adj R^2^ = 0.8761) further supported the model’s importance.

$$ {\text{R}}^{{2}} = {94}.{37}\%;\quad {\text{adjR}}^{{2}} = {87}.{61}\%. $$

The polynomial model equation suggested to estimate the ideal levels of the variables for PGA yield after the ANOVA statistical test was done, revealed that, the first order models for PGA production were satisfactory. The equation could be written as:

$$ \begin{aligned} {\text{PGA}}\;{\text{production}}\;{\text{S}}  = - 6.36 + 2.487\;{\text{pH}} + 0.0270\;{\text{glutamic}}\;{\text{acid}} \\  \quad + 0.0854\;{\text{yeast}} - 0.044\;{\text{incubation}}\;{\text{time}} \\  \quad - 0.0425\;{\text{temperature}} - 0.102\;{\text{inoculum}}\;{\text{size}}. \\ \end{aligned} $$

The points in the normal probability plots indicated the existing observed data, while the straight line in the plot represented the mathematical regression equation that established the predicted data. The residuals, or the difference between the observed and predicted data, were regarded as normally distributed, as presented in Fig. 2b and d, the points typically composed a line correspondent to the regression line.

### Purification of PGA

Extracellular PGA was released by both *Bacillus licheniformis* and *Bacillus subtilis* (ATCC6633). Three processes comprised the purification procedure of PGA yield: centrifugation was used to get rid of the bacterial cells, four liters of cold ethanol were used to sediment the polymer from the cell-free supernatant, and dialysis against distilled water was done to eliminate any possible contaminants.

## Characterization of PGA

### Fourier transform infrared spectroscopy (FTIR)

**Fig. 3:**
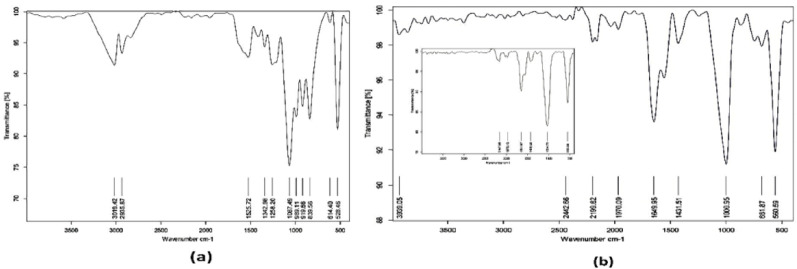
Infrared spectra of **a** Standard PGA and **b** PGA from *Bacillus licheniformis*. The inset shows the FTIR spectrum of standard PGA from *Bacillus subtilis* (ATCC6633)

FTIR was employed to assess the characteristics of the released PGA by *Bacillus licheniformis* and *Bacillus subtilis* (ATCC6633). Figure 3 showed the spectra of the two bacilli under investigation in comparison to the standard PGA. The spectra showed a strong distinctive C-N absorption peak at 1000–1068 cm^−1^, a characteristic amide peak at 1650 cm^−1^ (high amide absorption), and another peak at 1390–1450 cm^−1^ that corresponded to carbonyl absorption (C=O). When compared to the standard spectrum of PGA, the spectra verified that the products of *Bacillus subtilis* (ATCC6633) and *Bacillus licheniformis* had the unique carbonyl, amide, and other groups in PGA.

### Thin layer chromatography (TLC)

**Fig. 4:**
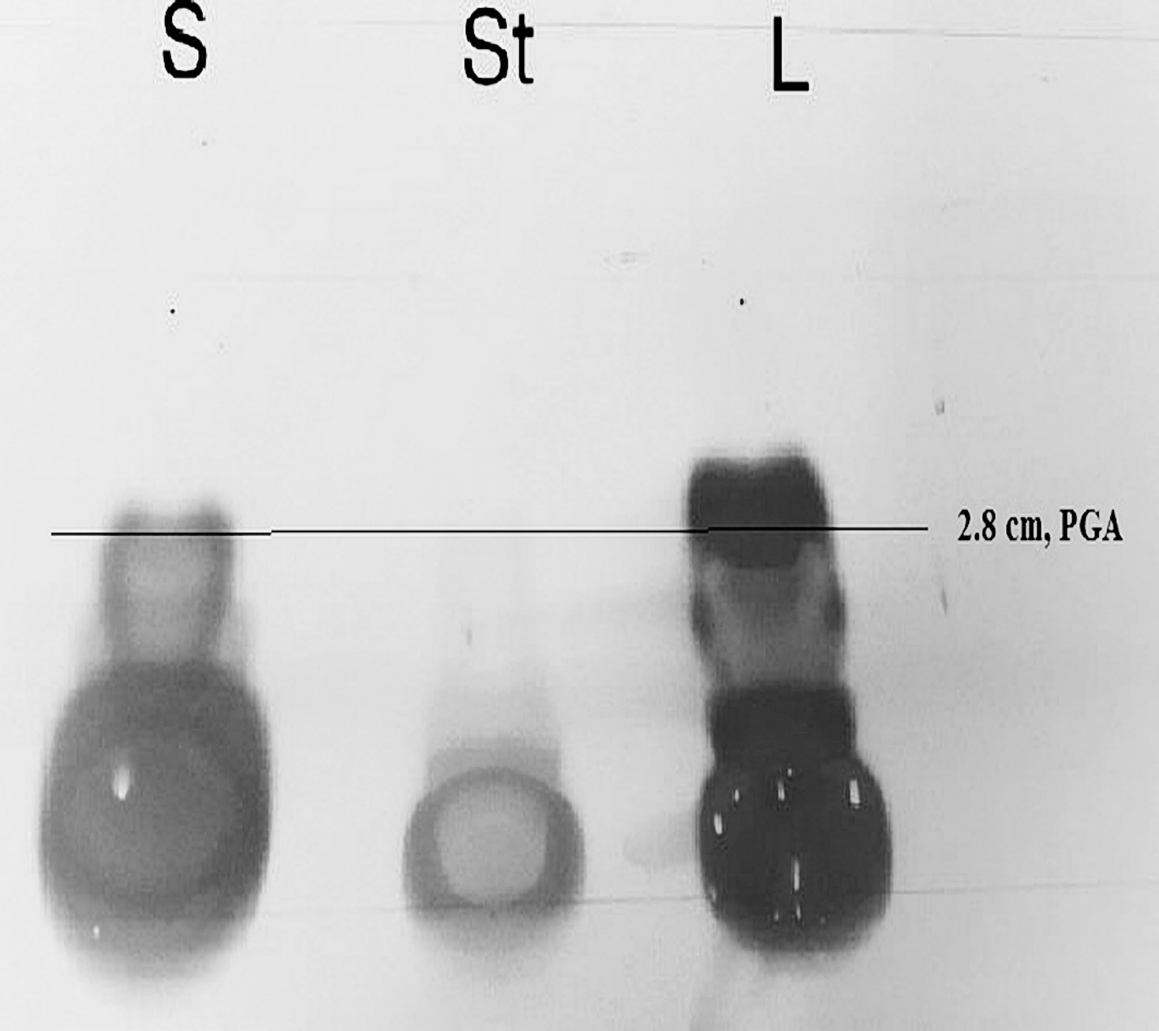
Amino acid analysis of γ-PGA by TLC method (St: γ-PGA standard control, S: PGA produced from *Bacillus subtilis* (ATCC6633), L: PGA produced from *Bacillus licheniformis*)

TLC was utilized to define the amino acid in the PGA synthesized by the *bacilli* in the current study. The separation concept of TLC is reliant on the compound’s relative affinities for the stationary and mobile phases. The current investigation assessed the amino acid available in the product released by the *bacillus* species. Figure 4 represented the 3 distinctive spots of the amino acid (glutamic acid) generated by *B. subtilis* (ATCC6633) and *B. licheniformis*. The distances traveled by the compounds were found to be 2.5 cm and 2.8 cm for the *Bacillus subtilis* (ATCC6633) and *Bacillus licheniformis,* respectively. The distance traveled by the glutamic acid standard (st) was measured to be 3cm. The acquired spots’ color was contrasted with the standard PGA color. The solvent was developed to 8.5 cm on the TLC plate. The retardation factor (R_f_) of the standard control was 0.35. *Bacillus subtilis* (ATCC6633) and *Bacillus licheniformis* R_f_ were 0.3 and 0.33, respectively. The existence of PGA was verified by comparing the retardation factors (R_f_) of each compound to that of the standard PGA.

### Liquid chromatography electrospray ionization tandem mass spectrometry (LC–ESI–MS)

LC–MS was utilized to verify the synthesis of PGA. Molecular ion peak at 274 m/z was present in the product of both *Bacillus subtilis* (ATCC6633) and *Bacillus licheniformis* as well as the standard PGA, suggesting that both *bacilli* produced and released PGA into the fermentation medium (figure S1 in SI file).

### Amino acid analysis

HCl extraction followed by injection in HPLC was used to measure the glutamic acid content of PGA. The amino acid composition of PGA generated by *Bacillus subtilis* (ATCC6633) and *Bacillus licheniformis* was presented in Table S2 (in SI file). Other amino acid contents were found with much lower than those of glutamic acid. This finding suggested that glutamic acid (with concentration of 508.70 mg/g, and 403.97 mg/g for PGA produced from *B. licheniformis* and *B. subtlis*, respectively) was the primary component of the resulting polymer.

### Effect of low levels of Gamma irradiation on the produced PGA

**Fig. 5:**
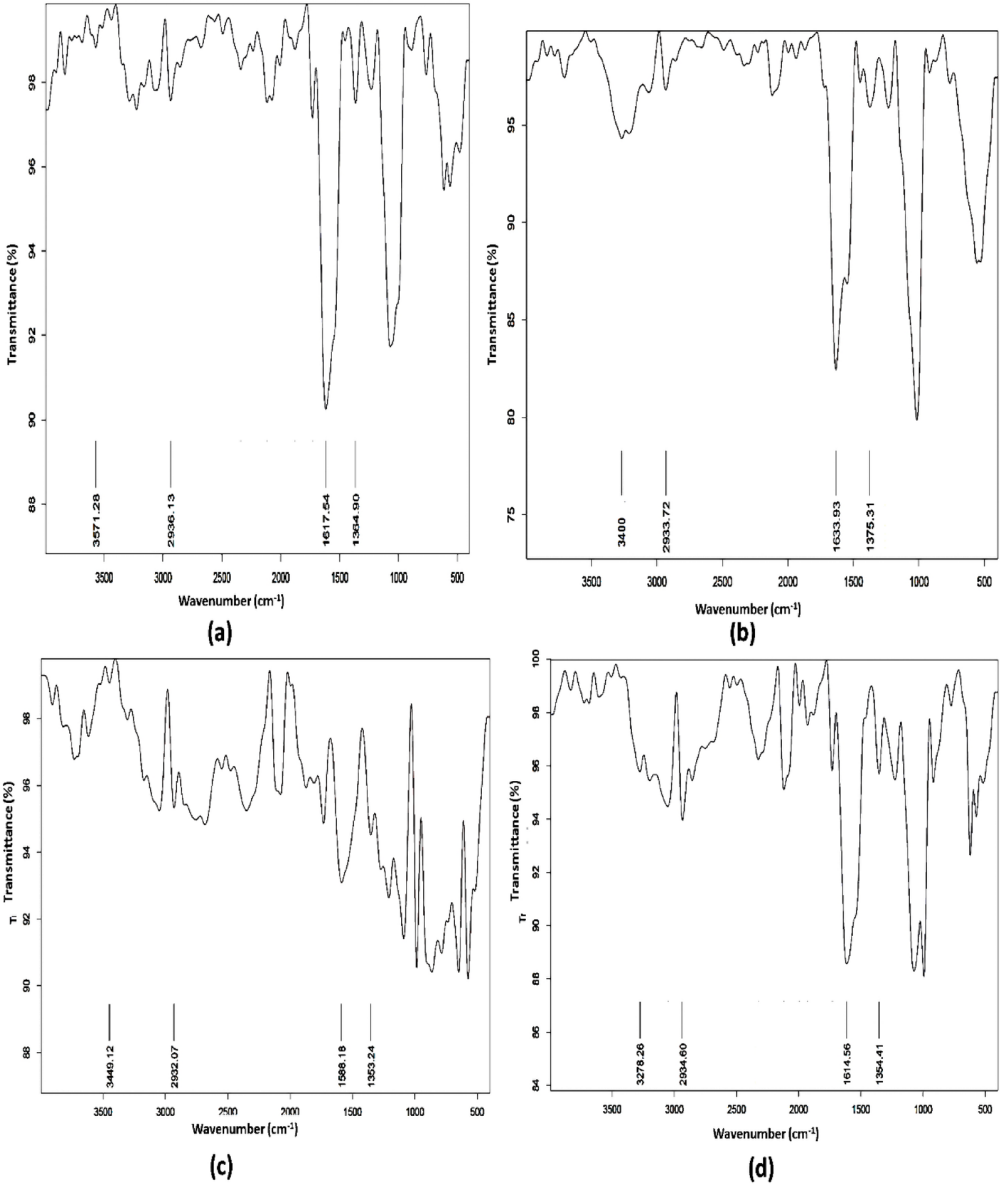
FTIR of polyglutamic acid produced from *Bacillus licheniformis* for non-irradiated PGA (**a**), PGA subjected to 2 Gy gamma irradiation (**b**), PGA subjected to 7.5 Gy gamma irradiation (**c**) and PGA subjected to 70 Gy gamma irradiation (**d**)

Upon subsequent exposure to gamma radiation, FTIR spectroscopy was utilized for its useful identification of the polymers’ characteristics. The variations in peak intensities were linked to the relative increase or decrease in the intensity of the characteristic bands of the functional groups in PGA. The spectra revealed the existence of PGA-specific bands at 3400 cm^−1^, which corresponded to the carbonyl group’s O–H. The distinctive aliphatic N–H stretching was responsible for the peak at 2935 cm^−1^, whereas the amide and C=O groups were expressed by the absorption bands at (1590–1630) cm^−1^ and (1355–1375) cm^−1^, respectively. There were no observable changes in the intensity or location of the distinctive FTIR peaks of PGA followed by gamma irradiation. This demonstrated that neither *Bacillus licheniformis* (Fig. 5) nor common *Bacillus subtilis* (ATCC 6633) (Figure S2 in SI file) experienced any alterations in PGA characteristics at low doses of gamma irradiation.

**Fig. 6:**
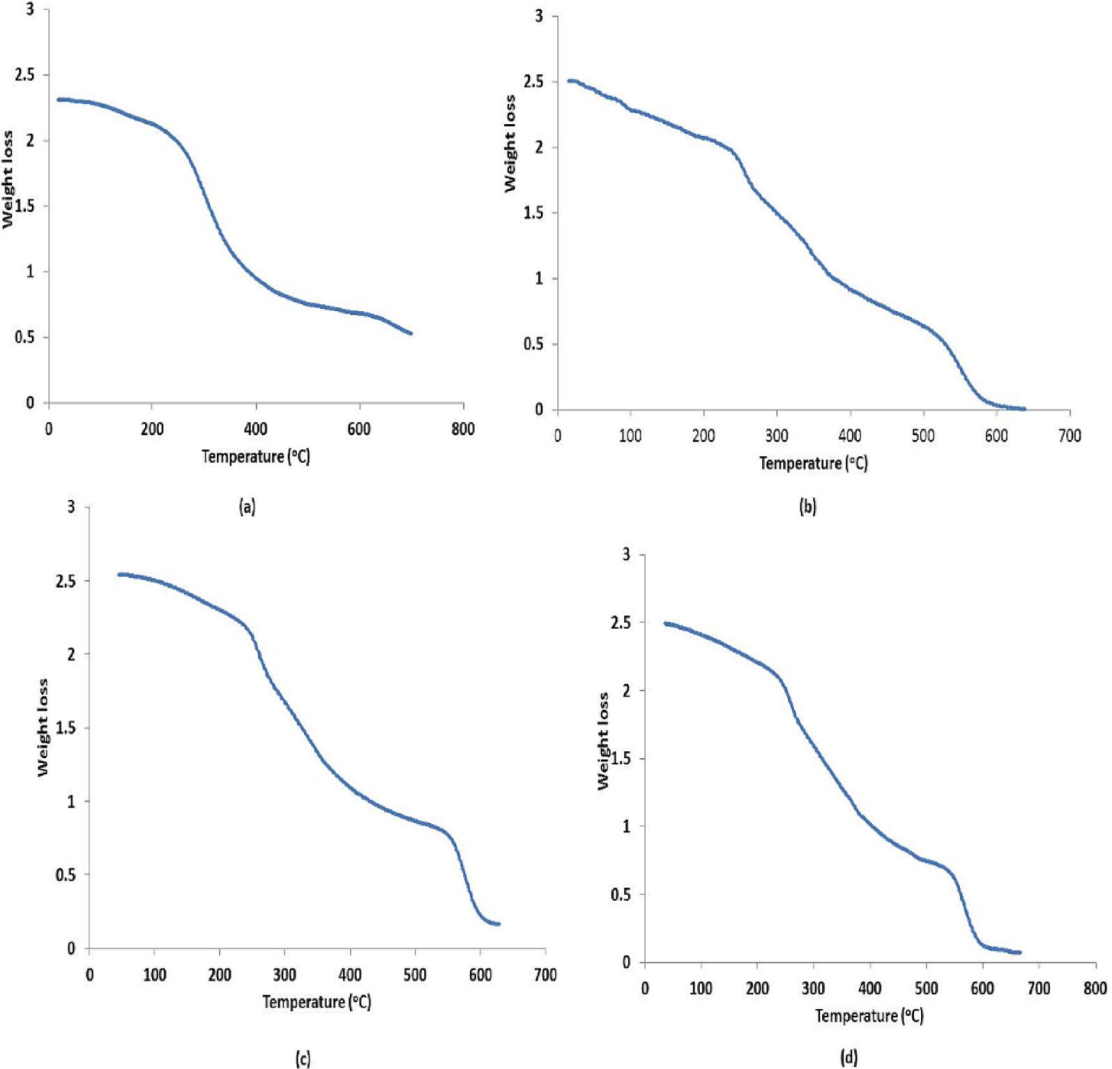
Thermal gravimetric profiles of PGA obtained from *Bacillus licheniformis* before exposure to gamma radiation (**a**) after exposure to 2 (**b**), 7.5 (**c**) and 70 (**d**) Gy of gamma radiation

The thermal gravimetric analysis (TGA) profile represented the response of PGA to increasing temperature. The TGA for PGA control showed a shoulder at 230°C that represented 0.3 weight loss and a second shoulder at 375°C with 1.3 weight loss (Fig. 6a). Figure 6b showed that gamma-irradiated (2 Gy) PGA had a shoulder at 220°C which represented 0.5 weight loss, and a second shoulder at 370 °C which demonstrated a weight loss of 1.5. At 7.5 Gy (Fig. 6c) the TGA profile of gamma irradiated PGA revealed weight loss of 0.3 and 1.3 at 225°C and 370 °C, respectively. While the gamma irradiated PGA at 70 Gy (Fig. 6d) exhibited a shoulder at 230 °C and 375 °C, which illustrated a weight loss in PGA of 0.4 and 1.4, respectively.

**Fig. 7:**
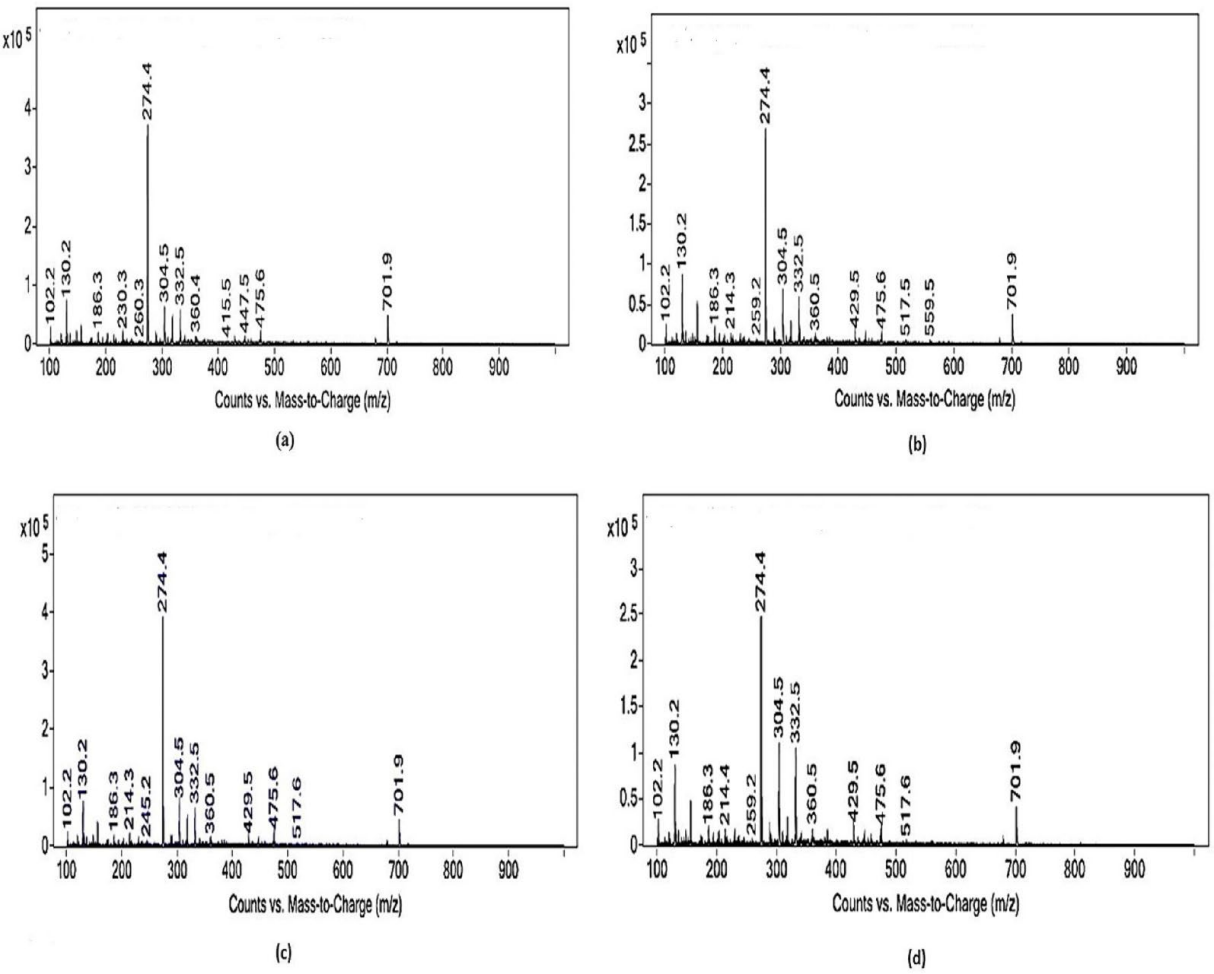
LC–MS of PGA produced from *Bacillus licheniformis* before exposure to gamma radiation (**a**) after exposure to 2 (**b**), 7.5 (**c**) and 70 (**d**) Gy of gamma radiation

The particle size of PGA control and gamma irradiated PGA at low doses, showed minor changes in DLS profile (5577.5, 4899.3, 4923.65, and 4950 nm, respectively) as shown in figure S3 (SI file). Additionally, LC–MS profile of PGA control and that of PGA at different low gamma radiation doses showed a molecular ion peak at 274 m/z (Fig. 7).

## Discussion

For decades, researchers have focused on the production of naturally occurring, environment friendly, and cost-effective biopolymers. Microbial biopolymers have been considered as viable remedies to human health problems. PGA is a polymer that is appealing due to its biocompatibility, biodegradability, non-toxicity, and lack of immunogenicity. Because of its distinct qualities, it is utilized in diverse industries, including pharmaceuticals, healthcare, and other essential fields (Elbanna et al. 2024). As a result, the current work aimed to investigate the production, optimization, and characterization of PGA derived from *Bacillus licheniformis*.

*Bacillus* species are the natural, predominant, and safe strains for PGA synthesis. *Bacilli* are commonly found in the soil (Li et al., 2022b). Sy et al. (2018) reported that a strong PGA-producing *Bacillus* sp. was isolated from soil. In the present investigation, soil samples were collected from different sectors in Egypt; the suspected *bacilli* colonies with the characteristic mucoid appearance on the selection medium were separated and analyzed for PGA production (Khadka et al. 2020). The most potent polymer producer (L isolate) was selected and molecularly identified by 16S rRNA as *Bacillus licheniformis* when compared to GenBank database in NCBI.

Screening the influencing factors for PGA production was performed by the Plackett–Burman layout to achieve the highest possible production rates. A number of variables were identified by this layout. The polymer productivity of *Bacillus licheniformis* was modified by adjusting of pH, incubation period, glutamic acid, and temperature. While in the case of standard *Bacillus subtilis* (ATCC6633), pH and yeast concentration influenced PGA optimization (Tork et al. 2015; Elbanna et al. 2024).

PH is a key component in PGA biosynthesis because it stimulates glutamate consumption, which results in higher PGA production. The current investigation indicated that the ideal pH for maximal PGA yield was 7 (± 0.2). pH has a substantial impact on PGA productivity, and it was shown that pH drop from 7.4 to 6.5 generated a 4-times increase in PGA output with *B. Iicheniformis* (Luo et al. 2016). Maximum PGA production was obtained with pH rising from 6.0 to 7.5, while the highest polymer generation obtained at pH 7 (Tork et al. 2015). Suitable fermentation factors, such as pH, oxygen content, temperature, and inoculum size, have been demonstrated to effectively boost PGA production (Zeng et al. 2014; Li et al. 2022b).

*Bacillus* species produce PGA outside of cells, making its purification easier. The standard procedure is to precipitate with organic solvents. Ethanol remains the recommended solvent for precipitating PGA from a cell-free media. PGA was then removed by centrifugation and dissolved in distilled water for dialysis. Lyophilization was the last stage to acquire the pure PGA (Lim et al. 2012).

*Bacillus licheniformis* is widely utilized for PGA production*.* Several studies optimized PGA production to achieve maximum yield*.* In the current work, by optimizing the conditions for PGA production by *B. licheniformis*, a yield of 24 g/L was obtained. A study on the local level applied the medium E for PGA production by *B. licheniformis*, resulted in a yield of 18.0 g/L at 72 h. Then the amount of PGA produced decreased after 72 h, due to nutrients depletion (Gomaa 2016). Thapa et al. 2021 claimed that *Bacillus licheniformis* KK7-12 was a potent PGA producer. Employing different conditions for bacterial growth such as incubation period, temperature, pH, and NaCl concentration affected PGA productivity and gave a yield of 22 g/L. Xavier et al. 2020 utilized the cost effective regional substrate (rice bran and casein peptone), pH of 7.5, and incubation temperature of 37 °C for 48 h to produce a yield of 8.2 g/L of PGA. Ebrahimzadeh Kouchesfahani et al. 2023 optimized the bioreactor conditions using the batch fermentation of *Bacillus licheniformis* ATCC 9945^a^. Under optimal conditions 15.5 g/L PGA was retrieved. Hence, under the stated optimized conditions, our local strain succeeded in producing a higher yield of PGA compared to other reported strains.

PGA was characterized and assessed utilizing Fourier transform infrared spectroscopy (FTIR) (Chettri et al. 2016). The present investigation findings are in accordance with (Ho et al. 2006), who reported that PGA exhibited a weak C=O absorption at (~ 1394–1454) cm^−1^, a strong hydroxyl absorption at (3400–3450) cm^−1^, and a distinct aliphatic N–H stretching between (2900–2800) cm^−1^, (1600–1660) cm^−1^, and (1390–1450) cm^−1^ for amide and C=O groups, respectively. The current study findings were similarly consistent with those published by Xi et al. 2022, Li et al. 2022c.

TLC analysis is a technique applied for determining the purity of the tested compounds. TLC detection of only glutamic acid is attributed to the purity of the polymer (Bheem 2019). According to Thapa et al. (2021), the distance traveled by the PGA samples was 3.2 cm, while that of the solvent was 12.5 cm on the TLC plate. Similarly, the distance traveled by positive control glutamic acid was discovered to be 3.1 cm. The retardation factor of the standard control was measured to be 0.25, and that of PGA released by the tested bacteria was measured to be 0.27. Comparing the retardation factor of the samples with other reports confirmed that the compounds generated were indeed PGA (Song et al. 2019). These findings are in harmony with the current investigation, which found that the distances developed by the tested compounds produced by* Bacillus subtilis* (ATCC6633) and *Bacillus licheniformis* were found to be 2.5 cm and 2.8 cm*,* respectively. The standard control’s retardation factor (R_f_) was 0.35, whereas *Bacillus subtilis* (ATCC6633) and *Bacillus licheniformis* had R_f_ of 0.3 and 0.33, respectively (Fig. 4). This indicated that the tested compound produced by both *bacilli* was PGA.

Liquid chromatography-mass spectrometry (LC–MS) is an advanced technique for analyzing new and known compounds, as well as understanding the chemical characteristics and structure of different molecules. It is highly beneficial for evaluating multiple components and shows improved sensitivity and selectivity (Mukherjee 2019). LC-ESI MS primarily recognized glutamic acid polypeptides in mixtures (Tork et al. 2015). The current study data revealed molecular ion peak at 274 m/z in the spectra of the tested compounds similar to the peaks of the standard PGA. This indicated that both *Bacillus subtilis* (ATCC6633) and *Bacillus licheniformis* biosynthesized and released PGA into fermentation medium. This finding is in harmony with (Li et al. 2022c; Kino et al. 2009) who discovered that all LC–MS peptide fragments were formed of glutamic acid.

The amino acid analysis by HPLC was used to determine the glutamic acid content of the polymer. In comparison to other trace amino acids, the current analysis confirmed that glutamic acid was the most abundant component in the formed polymer. *Bacillus licheniformis* isolated from the soil produced polyglutamic acid with a greater glutamic acid concentration than standard *Bacillus subtilis* (ATCC6633) (Table S2). Kubo et al. (2021) carried out a similar investigation and found that the PGA standard contains a small number of other amino acid impurities, although in less amounts compared to glutamic acid.

Gamma irradiation can cause changes in the molecular weight of polymers by either decreasing or increasing the molecular weight due to chain scission or cross-linking, depending on the applied dose (Aouat et al. 2019). According to Vasile et al. (2022), disproportionality, hydrogen elimination, rearrangement, degradation, and/or synthesis of new bonds can occur when polymeric materials (polylactic acid-based blends) are exposed to ionizing radiation (γ-rays, X-rays, accelerated electrons, etc.). It was found that the polylactic acid exhibited slight structural and property changes after being exposed to a dose of 10–20 kGy of gamma radiation (Piri et al. 2011; Vasile et al. 2022). Additionally, no observable alterations in the structure of polylactic acid were found, particularly at low doses of radiation (Bednarek et al. 2020). The current study results demonstrated that the peaks which corresponded to the PGA functional groups remained unchanged following exposure to low levels of gamma radiation (2.5, 7.5, and 70 Gy), which are the lowest, highest, and cumulative radiation doses for cancer patients undergoing radiotherapy, respectively, as reported by Tao et al. (2023).

Thermal properties of PGA were measured using thermal gravimetric analysis by heating the obtained polymer at 25 °C/min. The current study result showed a change in the first phase observed for PGA exposed to gamma-radiation, which is an indication of loss of moisture content. Maghrawy et al. 2024 reported that the exopolysaccharide, which is a natural biopolymer produced by microorganisms showed similar phases of change in the thermal gravimetric analysis as PGA. The results of the present work revealed that the gamma irradiated polymer showed decomposition temperatures of 220–370 °C, 225–370 °C and 230–375 °C for radiation levels of 2, 7.5 and 70 Gy, respectively. Xavier et al. 2020 reported that the decomposition temperature of PGA was 209–299.7 °C. This indicated that the produced PGA exhibited thermal stable bonding when exposed to gamma radiation doses similar to those of cancer patients under radiotherapy. According to dispersion of particles at the time of experiment, minor changes were detected in DLS profile, whereas no changes were found in LC–MS, FTIR and TGA profiles after PGA exposure to low doses of gamma radiation. This finding makes PGA the drug carrier of choice for chemotherapeutic agents administered to cancer patients undergoing radiation therapy. To the best of the researchers’ information, no found articles have examined how gamma irradiation influenced PGA. We came to the conclusion that *Bacillus licheniformis* can produce PGA after optimization of the appropriate parameters, including temperature, pH, glutamic acid content, and incubation time. The fact that PGA is a durable polymer that remains unaltered after exposure to low levels of gamma irradiation up to 70 Gy is a promising outcome. This indicated that PGA can be the most convenient drug carrier for chemotherapeutic agents given to cancer patients receiving radiotherapy. It can be advised, that under right optimization conditions, *B. licheniformis* could be a potential strain for commercially producing PGA for industry after scaling up research.

## Electronic supplementary material

Below is the link to the electronic supplementary material.

Supplementary Material 1

Supplementary Material R2

## Data Availability

The published article contains all of the data generated or analyzed during this investigation.

## Abbreviations

ATCC: American type culture collection
DLS: Dynamic light scattering
FTIR: Fourier transform infrared spectroscopy
Gy: Gray
LC- MS: Liquid chromatography mass spectrometry
NCBI: National Centre for Biotechnology Information
OD: Optical density
PB: Plackett–Burman
PGA: Polyglutamic acid
TGA: Thermal gravimetric analysis
