# Supplementary material for: Production and optimization of polyglutamic acid from Bacillus licheniformis: effect of low levels of gamma radiation
Source: AMB Express. 2025 Jun 18;15:93. doi: 10.1186/s13568-025-01897-3 (PMC12176725; doi:10.1186/s13568-025-01897-3)
Supplement: Supplementary file 1 — Supplementary Material 1 [file 13568_2025_1897_MOESM1_ESM.docx]

**Table S1:** Optical density (O.D.) obtained for the runs of the Plackett-Burman layout

| Run | **pH** | **glutamic acid** | **yeast** | **incubation time** | **temperature** | **inoculum size** | **O.D. of PGA by *B. subtilis*** | **O.D. of PGA by *B .*licheniformis** |
| --- | --- | --- | --- | --- | --- | --- | --- | --- |
| 1 | 3 | 40 | 2 | 3 | 25 | 2.0 | 0.043 ± 0.2 | 0.042 ± 0.1 |
| 2 | 7 | 2 | 40 | 7 | 25 | 2.0 | 1.189 ± 0.2 | 0.241 ± 0.1 |
| 3 | 3 | 2 | 2 | 7 | 37 | 2.0 | 0.019 ± 0.1 | 0.015 ±0.2 |
| 4 | 7 | 40 | 40 | 3 | 37 | 2.0 | 1.370 ± 0.2 | 2.736 ± 0.2 |
| 5 | 7 | 2 | 40 | 3 | 25 | 0.5 | 1.357 ± 0.1 | 1.608 ± 0.1 |
| 6 | 3 | 2 | 40 | 7 | 37 | 0.5 | 0.324 ± 0.1 | 0.258 ±0.1 |
| 7 | 7 | 2 | 2 | 3 | 37 | 2.0 | 0.917 ± 0.2 | 1.206 ± 0.2 |
| 8 | 7 | 40 | 2 | 7 | 25 | 0.5 | 1.103 ± 0.2 | 1.166 ± 0.2 |
| 9 | 3 | 2 | 2 | 3 | 25 | 0.5 | 0.053 ± 0.1 | 0.042 ± 0.1 |
| 10 | 7 | 40 | 2 | 7 | 37 | 0.5 | 0.973 ± 0.2 | 1.401 ± 0.1 |
| 11 | 3 | 40 | 40 | 7 | 25 | 2.0 | 0.289 ± 0.2 | 0.220 ± 0.2 |
| 12 | 3 | 40 | 40 | 3 | 37 | 0.5 | 0.298 ± 0.1 | 1.324 ± 0.2 |

O.D. of 2 mg/ mL standard PGA = 0.228

**Table S2: Amino acids sequencing of PGA purified from *Bacillus licheniformis*** **and *B. subtilis* (ATCC6633)**

| Amino Acid | Conc. (mg/g) /  *B. licheniformis* | Conc. (mg/g) /  *B. subtilis* |
| --- | --- | --- |
| Aspartate | 15.16 | 22.43 |
| Glutamate | **508.70** | **403.97** |
| Serine | 6.68 | 10.41 |
| Histidine | 6.12 | 8.55 |
| Glycine | 29.09 | 71.07 |
| Threonine | 4.62 | 0.00 |
| Arginine | 10.47 | 19.30 |
| Alanine | 16.81 | 29.52 |
| Tyrosine | 3.48 | 0.00 |
| Cystine | 0.00 | 0.00 |
| Valine | 7.25 | 7.26 |
| Methionine | 1.41 | 0.00 |
| Phenylalanine | 5.84 | 6.46 |


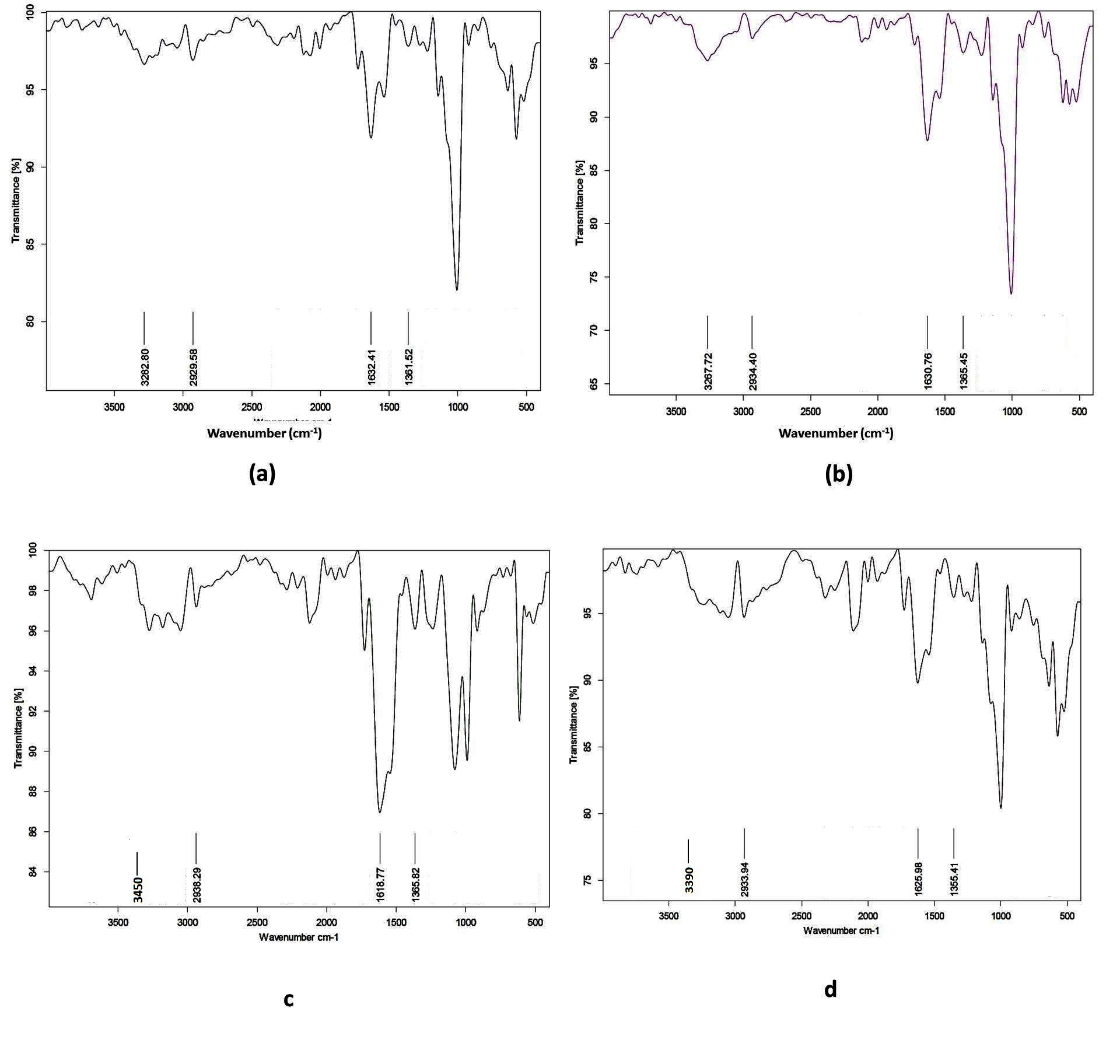
**Figure S1:** FTIR of polyglutamic acid produced from *Bacillus subtilis* (ATCC6633) for non-irradiated PGA (**a**), PGA subjected to2 Gy gamma irradiation (**b**), PGA subjected to 7.5 Gy gamma irradiation (c) and PGA subjected to 70 Gy gamma irradiation (d).


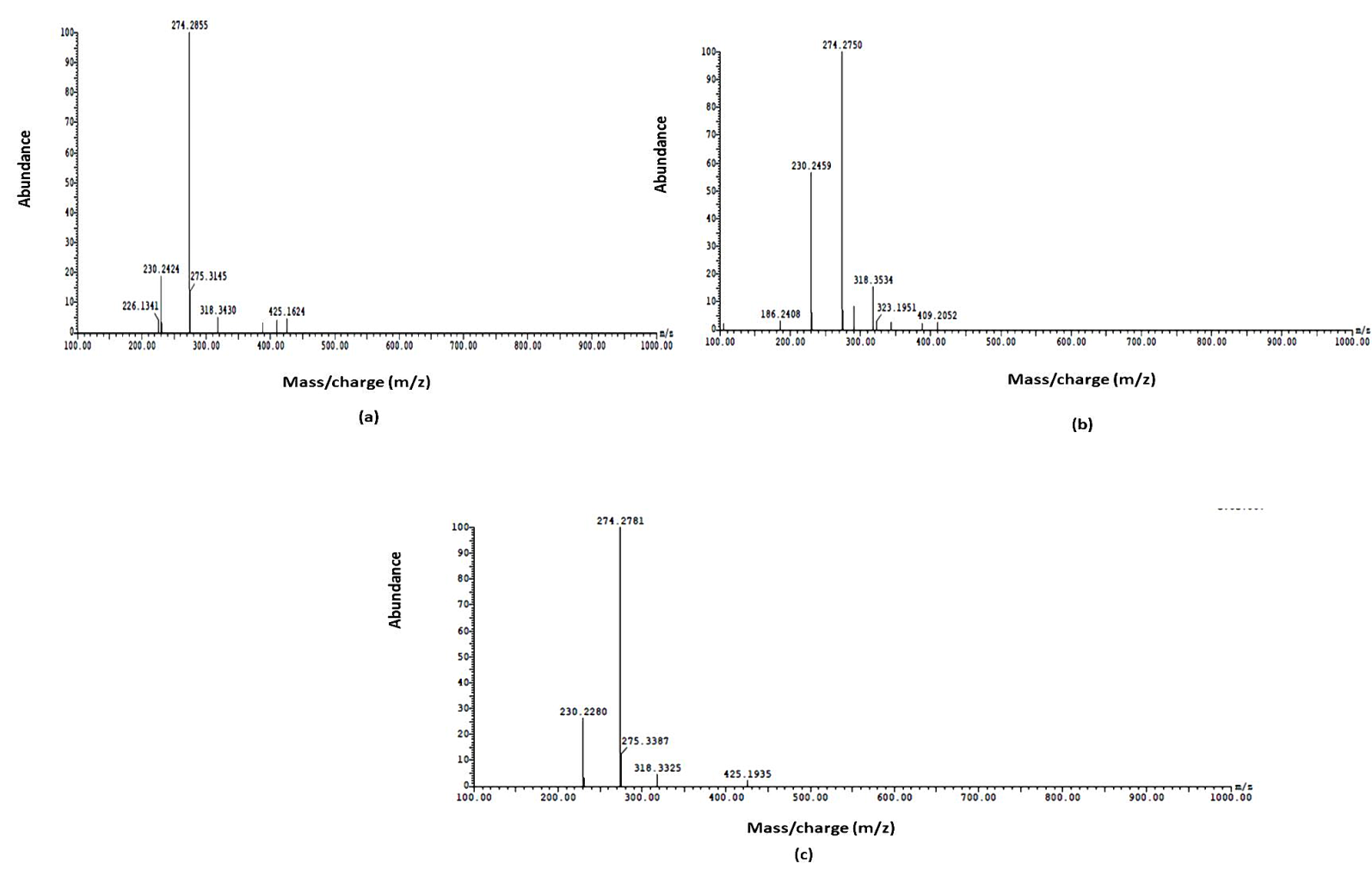


**Figure S2:** Characterization of the PGA produced by *Bacillus* spp. evaluated by LC-MS Polyglutamic acid standard (**a**); *Bacillus subtilis* (ATCC6633) (**b**); *Bacillus licheniformis* (**c**).


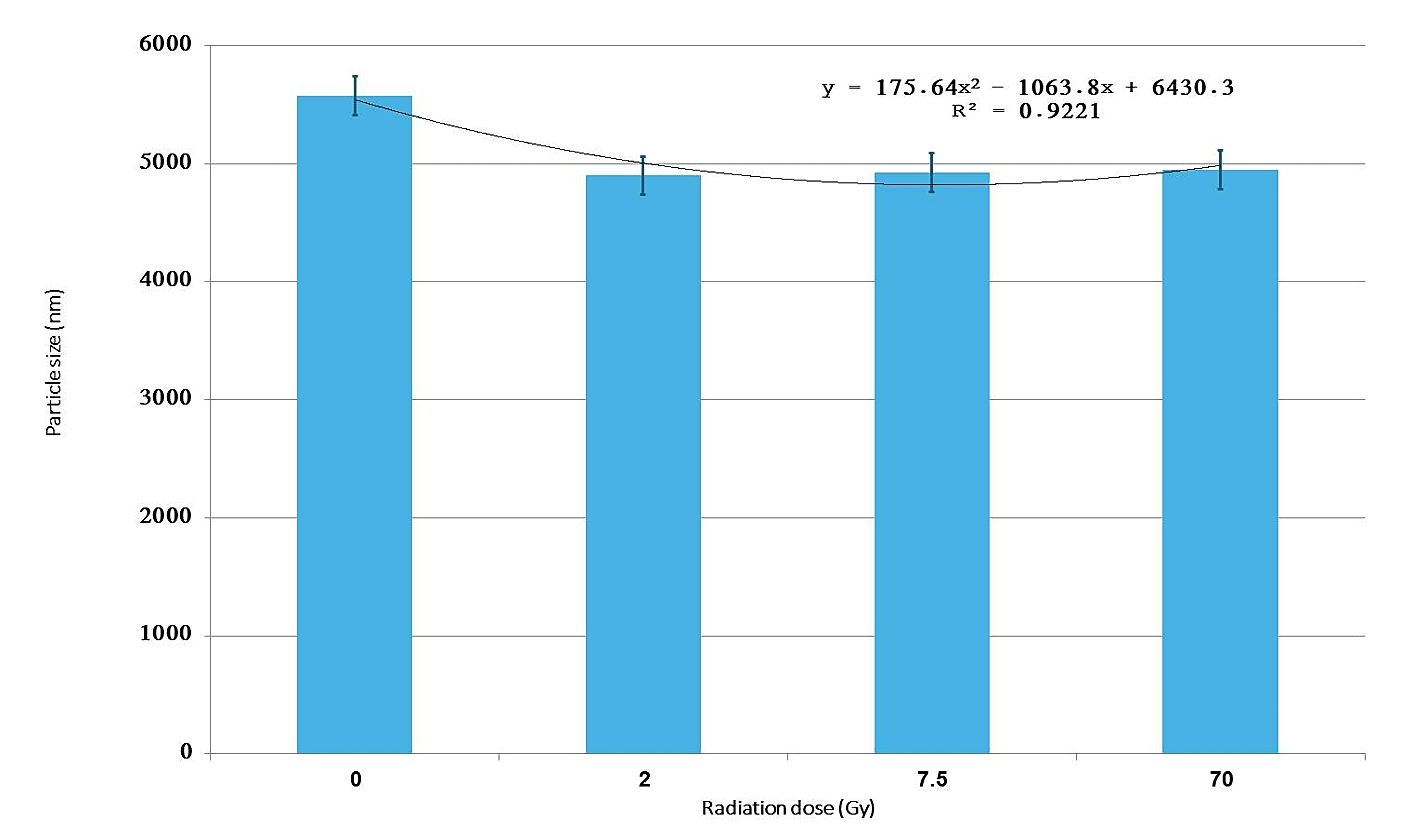


**Figure S3:** Particle size of PGA produced from *Bacillus licheniformis* before exposure to gamma radiation **(a)** after exposure to 2 **(b)**, 7.5 **(c)** and 70 **(d)** Gy of gamma radiation.
